# RETRACTION: LncRNA NEAT1 Targets MiR‐125/ADAM9 Mediated NF‐κB Pathway in Inflammatory Response of Rosacea

**DOI:** 10.1111/srt.70185

**Published:** 2025-05-15

**RETRACTION**: S. Xu, and W. Dong, “LncRNA NEAT1 Targets MiR‐125/ADAM9 Mediated NF‐κB Pathway in Inflammatory Response of Rosacea,” *Skin Research and Technology* 30, no. 7 (2024): e13630, https://doi.org/10.1111/srt.13630.

The above article, published online on 10 July 2024 in Wiley Online Library (wileyonlinelibrary.com), has been retracted by agreement between *Skin Research and Technology* and John Wiley & Sons Ltd. The article has been accepted for publication following an inadequate peer review process. Following publication of the article, several flaws and inconsistencies between results presented and methodology described were found. Furthermore, the article lacks important information to interpret and reproduce the findings. The material provided by the authors upon request was insufficient to address the concerns. Therefore, the decision to retract this article was taken as the editors consider its conclusions invalid. The authors have been informed of the decision of retraction but not available for a final confirmation.

